# Long COVID Research, 2020–2024: A PubMed-Based Bibliometric Analysis

**DOI:** 10.3390/healthcare13030298

**Published:** 2025-02-01

**Authors:** Cristina Honorato-Cia, Elena Cacho-Asenjo, Antonio Martinez-Simon, Irene Aquerreta, Jorge M. Núñez-Córdoba

**Affiliations:** 1 Department of Anaesthesia and Critical Care, Clínica Universidad de Navarra, 31008 Pamplona, Spain; 2 IdiSNA, Navarra Institute for Health Research, 31008 Pamplona, Spain; 3 Pharmacy Service, Clínica Universidad de Navarra, 31008 Pamplona, Spain; 4 Research Support Service, Central Clinical Trials Unit, Clínica Universidad de Navarra, 31008 Pamplona, Spain; 5 Institute of Data Science and Artificial Intelligence, University of Navarra, 31009 Pamplona, Spain; 6 Department of Health Sciences, Public University of Navarra, 31008 Pamplona, Spain

**Keywords:** long COVID, post COVID-19, chronic COVID-19, SARS-CoV-2, post-acute sequelae, COVID-19, bibliometrics, research profile

## Abstract

Long COVID is a SARS-CoV-2 infection-associated chronic condition with great potential to impact health and socioeconomic outcomes. The research efforts to face the challenges related to long COVID have resulted in a substantial amount of publications, which warrants the need for bibliometric profiling. This is a large-scale PubMed-based bibliometric analysis of more than 390,000 COVID-19 publications. The overall aim was to update the profile of long COVID publications in comparison with the rest of the COVID-19 scientific literature through December 2024. The estimated proportion of long COVID publications was relatively low (2.3% of all COVID-19 publications), although the cumulative frequency (n = 8928) continues to pose a challenge for proper information management. Currently, “treatment” and “mechanism” appear to be the most predominant research topics in the long COVID literature. Interestingly, this evaluation revealed a distinctive profile of the long COVID literature, with a clear preponderance of “case report” and “mechanism” research topics when compared with other COVID-19 publications. This evaluation also identified and ranked the most prolific scientific journals in the production of long COVID-related publications. This study may improve the visibility of long COVID research and contribute to the management of the growing scientific knowledge on long COVID.

## 1. Introduction

The cumulative total number of reported COVID-19 cases exceeds 776 million worldwide [1]. Anyone exposed to SARS-CoV-2 can suffer post-acute sequelae of SARS-CoV-2 infection (PASC), also known as long COVID, regardless of age, sex, race, or severity of original symptoms [2,3,4]. The National Academies of Sciences, Engineering, and Medicine (NASEM) has defined long COVID as an infection-associated chronic condition that occurs after SARS-CoV-2 infection and is present for at least 3 months as a continuous, relapsing and remitting, or progressive disease state that affects one or more organ systems [5].

The impact of long COVID on quality of life seems to be worrisome [6]. In addition to the health impact of long COVID in patients, the social and economic consequences of this condition are also of concern [7].

Progression in knowledge of the mechanisms, prevention, diagnosis, and treatment related to long COVID is underway, although major challenges remain [8]. Several priority areas for further research on long COVID have been suggested. These include topics such as vaccines, next-generation clinical trials, long COVID at extremes of age, optimizing rehabilitation protocols, and optimizing health services [9].

The scientific efforts to face the challenges related to the COVID-19 pandemic have resulted in an overwhelming amount of research literature on one single disease in a fairly short time frame from the pandemic’s onset in 2020. By December 2024, the COVID-19 literature available in PubMed exceeded 390,000 publications. Several bibliometric studies have explored COVID-19 research trends and patterns, thus contributing useful information to the management of this unprecedented wave of publications. Overall, COVID-19 bibliometric analyses range from globally focused studies [10,11,12] to those specifically focused on geographical areas [13,14,15,16], research topics [17,18,19], or medical fields [20,21,22,23,24,25,26,27,28,29,30,31,32,33,34,35].

The description of the patterns of the long COVID research literature is also gaining interest. However, valuable bibliometric studies related to long COVID publications are yet scarce and limited to the long COVID research domain [36,37,38,39] or subdomain [40,41,42] without comparing with other COVID-19 publications.

The main purpose of this bibliometric analysis was to quantify the proportion of long COVID scientific publications among the entire COVID-19 literature from the beginning of the COVID-19 pandemic to the end of 2024. Second, we aimed to describe trends and patterns in long COVID publications, and to compare research topics between long COVID publications and the remaining of COVID-19 research. Finally, we aimed to identify the most prolific journals in the production of the long COVID literature. This study reveals a distinctive profile of long COVID publications compared with the other COVID-19 literature as a whole. This is a useful information for researchers, clinicians, and policymakers who need to identify what long COVID research topics are highlighted and how these change over time.

## 2. Materials and Methods

### 2.1. Study Design

This is a large-scale bibliometric analysis of more than 390,000 COVID-19 publications. This evaluation is part of the Covid Content Curation Project (Research Grant Numbers: 0011-3638-2020-000001 and 49-2022, Health Department of Navarra Government, Spain), an ongoing research project designing an artificial intelligence platform for grading the relevance of COVID-19 scientific publications for decision making.

### 2.2. Selection of COVID-19 Publications

The information source for all COVID-19 publications was the PubMed database. No restrictions on language or journal scientometric index were applied. Duplicate publications and publications from the preprint servers for specialized research networks (SSRN), physics and other sciences (arXiv), chemistry (ChemRxiv), biology (bioRxiv), and health sciences (medRxiv) were excluded. The remaining exclusion criteria were publication before 1 March 2020 (the World Health Organization COVID-19 pandemic declaration was on 11 March 2020) or after 31 December 2024, and those with any missing date data.

### 2.3. Characterization of COVID-19 Publications

All COVID-19 publications were classified as long COVID or other COVID-19 publications, and seven research topic categories (“mechanism”, “transmission”, “diagnosis”, “treatment”, “prevention”, “case report”, and “forecasting”) were defined following updated filter search strategies that were documented in the PubMed User Guide [43]. Additional journal information included the subject category and journal impact factor (JIF) that was obtained from the Journal Citation Reports (JCR) dataset of Clarivate Analytics Web of Science.

### 2.4. Ethical Statement

Ethical approval was not essential for this study because it uses publicly available data from documents. This study is part of the Covid Content Curation Project, which has been approved by the Research Ethics Committee of the University of Navarra, Spain (Reference number: 2022.101).

### 2.5. Statistical Analysis

The two-proportions z-test was used to compare research topic categories of COVID-19 publications between the long COVID literature and the other COVID-19 literature. The Mann–Kendall test was used to evaluate the research topic trends over the study period. *p* values <0.05 were considered statistically significant. Statistical analyses were conducted using Stata 18 (StataCorp. 2023. Stata Statistical Software: Release 18. College Station, TX, USA: StataCorp LLC).

## 3. Results

**Figure 1:**
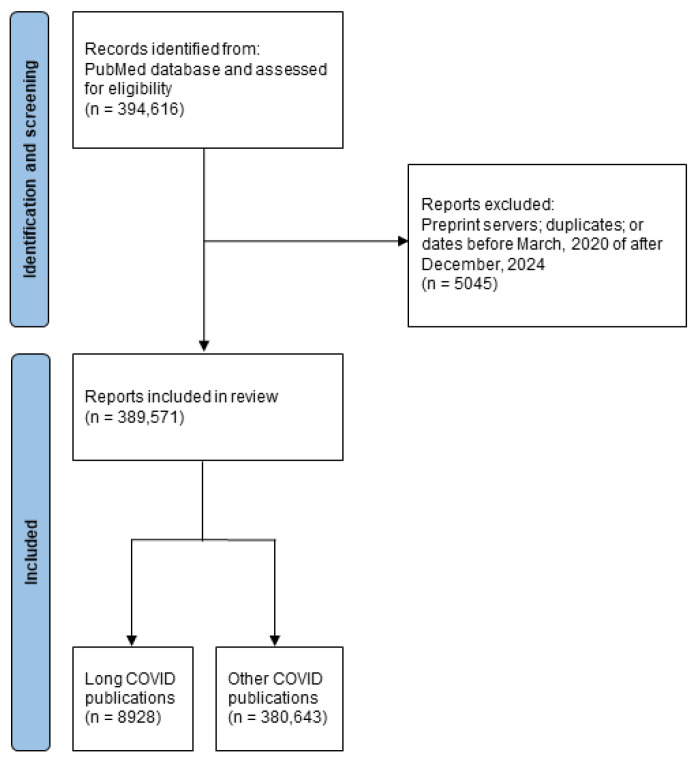
Flow diagram of study selection.

The data from a total of 394,616 COVID-19 publications were extracted from PubMed. After the exclusion criteria were applied (duplicate publications, publications from preprint servers, publications published before 1 March 2020 or after 31 December 2024, or with any missing date data), 389,571 COVID-19 publications were available for analysis (Figure 1).

**Figure 2:**
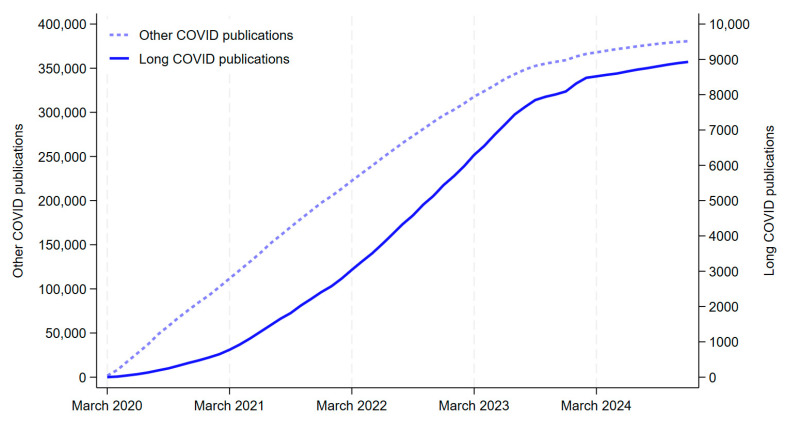
Cumulative number of COVID-19 publications, 2020–2024.

Figure 2 shows the cumulative number of long COVID publications and other COVID publications from March 2020 (the month when the World Health Organization declared the COVID-19 pandemic, 11 March 2020) to December 2024. The cumulative total of long COVID publications was 8928 (2.3% of all COVID-19 publications).

The predominant topics in long COVID publications were “treatment” (n = 4515 publications, 50.6%) and “mechanism” (n = 4279 publications, 47.9%) (Table 1). When compared with other COVID publications (n = 380,643), long COVID publications showed a significantly higher frequency in research topics dealing with “mechanism” (47.9% vs. 26.8%; *p* < 0.001), “case report” (39.4% vs. 20.9%; *p* < 0.001), “diagnosis” (39.6% vs. 28.4%; *p* < 0.001), and “treatment” (50.6% vs. 44.6%; *p* < 0.001). In contrast, the research topics “transmission”, “prevention”, and “forecasting” were less common in long COVID publications than in other COVID publications. The “transmission” theme was analyzed in 3.5% (n = 311) of the long COVID publications, whereas this topic was addressed in 9.9% (n = 37,659) of the other COVID publications. The differences between long COVID and other COVID publications in the “prevention” and “forecasting” topics were −5.4 percentage points (95% CI: −4.4 to −6.5; *p* < 0.001) and −3.0 percentage points (95% CI: −2.6 to −3.5; *p* < 0.001), respectively.

**Figure 3:**
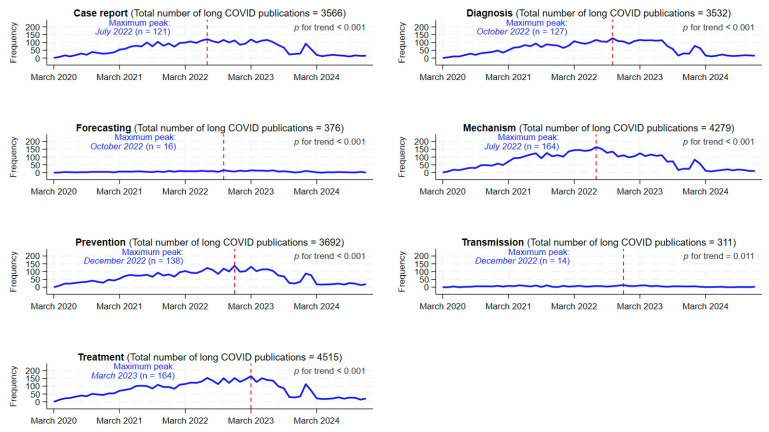
Time trends of long COVID publications according to research topics, 2020–2024. The red dashed vertical line indicates the month when the long COVID publication maximum peak occurred. *p* for trend values was calculated using the Mann–Kendall test (from onset to time of maximum peak).

The distributions of long COVID publications over time and by research topics are displayed in Figure 3. Interestingly, the topics “case report”, “diagnosis”, “mechanism”, “prevention”, and “treatment” showed a similar time pattern. This common pattern comprised a gradual upslope and a steeper downslope after the month with the highest frequency of publications. The maximum monthly frequency ranged from 14 publications for “transmission” to 164 publications for “mechanism” and “treatment”. The majority of research topics had the maximum monthly frequency of long COVID publications during the year 2022, with the exception of “treatment”, which had its maximum peak in March 2023. The earliest maximum peak (July 2022) was observed in the “case report” category. Overall, the frequencies of long COVID publications on “forecasting” and “transmission” appeared lower compared with the rest of the research topics and showed a relatively plateauing pattern.

**Figure 4:**
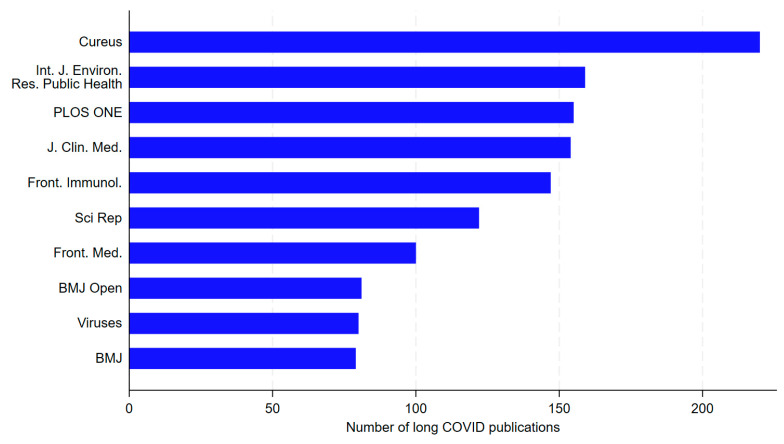
Top 10 journals with the highest frequency of long COVID publications, 2020–2024. BMJ: *The BMJ*; Front. Immunol.: *Frontiers in Immunology*; Front. Med.: *Frontiers in Medicine*; Int. J. Environ. Res. Public Health: *International Journal of Environmental Research and Public Health*; J. Clin. Med.: *Journal of Clinical Medicine*; Sci Rep: *Scientific Reports*.

During 2020–2024, a total of 2254 journals published at least one article on long COVID. The top ten most productive journals were *Cureus* (2023 JIF: 1.000; JIF quartile: 3; Category: “Medicine, general & internal”; n = 220 long COVID publications; predominant research topic: “case report”, 77.3%), *International Journal of Environmental Research and Public Health* (2021 IF: 4.614; JIF quartile: 2; Category: “Public, environmental & occupational health”; n = 159; predominant research topic: “treatment”, 49.1%), *PLOS ONE* (2023 JIF: 2.900; JIF quartile: 1; Category: “Multidisciplinary sciences”; n = 155; predominant research topic: “prevention”, 51.6%), *Journal of Clinical Medicine* (IF: 2023 JIF: 3.000; JIF quartile: 1; Category: “Medicine, general & internal”; n = 154; predominant research topic: “treatment”, 45.5%), *Frontiers in Immunology* (2023 JIF: 5.700; JIF quartile: 1; Category: “Immunology”; n = 147; predominant research topic: “mechanism”, 74.1%), *Scientific Reports* (2023 JIF: 3.800; JIF quartile: 1; Category: “Multidisciplinary sciences”; n = 122; predominant research topic: “mechanism”, 59.8%), *Frontiers in Medicine* (2023 JIF: 3.100; JIF quartile: 1; Category: “Medicine, general & internal”; n = 100; predominant research topic: “treatment”, 43.0%), *BMJ Open* (2023 JIF: 2.400; JIF quartile: 1; Category: “Medicine, general & internal”; n = 81; predominant research topic: “treatment”, 59.3%), *Viruses* (2023 JIF: 3.800; JIF quartile: 2; Category: “Virology”; n = 80; predominant research topic: “mechanism”, 75.0%), and *The BMJ* (2023 JIF: 93.700; JIF quartile: 1; Category: “Medicine, general & internal”; n = 79; predominant research topic: “mechanism”, 53.2%) (Figure 4).

## 4. Discussion

This bibliometric analysis provides an updated profiling of the long COVID research literature as of December 2024, in the context of an unprecedented bibliometric phenomenon due to the enormous wave of COVID-19 publications.

Several previous studies have contributed to the profiling of the long COVID literature, paying attention to specific components of the long COVID condition, such as sequelae [40,41] and treatment [42]. The scope of our analysis was inspired by studies that targeted a global coverage of the long COVID literature [36,37,38,39]. Jin et al. conducted a bibliometric analysis on long COVID based on 784 articles or reviews retrieved from Scopus limited to the years 2020 and 2021 [36]. The PubMed-based bibliometric evaluation carried out by Porter et al. included 5243 articles through November 2022 [37]. Liu et al. also performed a study through November 2022 but using the Web of Science Core Collection as the data source and including a total of 3633 publications [38]. Lai et al. also used data from the Web of Science Core Collection and limited the evaluation to the “article” and “review” categories through February 2023, finally including 1765 studies [39].

Our study updates and complements previous studies with an approach that includes a comparison between publications on long COVID (8928 publications) and the rest of the COVID-19 research literature (380,643 publications). This novelty may contribute to framing long COVID scientific production in a fuller context.

Previous studies have supposed that despite the important generation of the long COVID literature, the number of long COVID publications may be relatively negligible compared with the rest of the COVID-19 literature [37]. Our study tested this hypothesis after evaluating more than 385,000 publications and estimated that long COVID publications currently represent 2.3% of all COVID-19 publications. Even though this percentage may be considered low, the current long COVID literature involves almost 9000 publications, a number that is already significant and that presumably will remain growing for the foreseeable future.

Our evaluation revealed a distinctive profile of long COVID publications when compared with the other COVID-19 literature as a whole. Thus, the long COVID research literature clearly stood out in the “case report” and “mechanism” topics, based on both the statistical significance of the difference and the magnitude of the percentage point difference between the groups of publications. The preponderance of the “case report” topic seems reasonable due to the novelty and diverse expression of long COVID. The focus on mechanistic studies usually occurs at an early stage [44]. Interestingly, both “case report” and “mechanisms” topics were the earliest to reach the maximum peak of publications (July 2022). The proportions of the research topics “diagnosis” and “treatment” were also greater in long COVID publications. The maximum monthly frequencies seem to follow a quite logical sequence, being December 2022 for “diagnosis” and March 2023 for “treatment”, assuming that the maximum peaks could be a proxy of the time when the research community showed the greatest interest in a specific topic.

Our findings agree substantially with other studies identifying the most productive scientific journals on long COVID publications. Our top ten classification includes eight of the nine most prolific journals reported by Porter et al. [37], and eight of the top ten journals reported by Lai et al. [39]. Furthermore, our evaluation concluded with the same rankings for the top two journals reported by Porter et al. [37] (*Cureus* and *International Journal of Environmental Research and Public Health*). The overall consistency does not reduce remarkably even when compared with the journal rankings provided by further earlier studies [36], suggesting that the overall configuration of the group of leading journals on long COVID topics did not change considerably from the early stages of long COVID research.

To our knowledge, this is the largest bibliometric analysis to date on COVID-19, evaluating more than 390,000 COVID-19 publications (and more than 8900 long COVID publications). These results provide an overview of research into COVID-19 and long COVID, and identify research distribution over time, which may be of great relevance for the targeted formulation of health policies. Long COVID has the potential to represent a substantial global public health challenge, with multifaceted impacts on patients and healthcare systems worldwide [6,7]. Our study profiles significant research topics that encompass some of the most relevant issues that need to be faced to address the long COVID threat successfully. There is a critical need for a standardized definition to effectively diagnose long COVID-affected patients and evaluate prevention and treatment strategies. Several clinical or score-based definitions of long COVID have been identified [45], with differences in timing of onset and duration of symptoms. No pathognomonic symptoms or definitive and available biomarkers have been described. Among the most common symptoms of long COVID are fatigue, shortness of breath, and cognitive dysfunction [46]. Long COVID symptoms can be mild to severe, and they can wax and wane. Diagnosable conditions (new or worsening pre-existing conditions) include migraine, stroke, mood disorders, cardiovascular disease, arrhythmias, blood clots, interstitial lung disease, hypoxemia, chronic kidney disease, postural orthostatic tachycardia syndrome and other forms of dysautonomia, mast-cell activation syndrome, hyperlipidemia and diabetes, myalgic encephalomyelitis–chronic fatigue syndrome, lupus, Sjögren’s, rheumatoid arthritis, and other connective tissue diseases or autoimmune disorders. The NASEM has concerted efforts to improve the definition of long COVID [5].

Understanding of the pathobiology and pathophysiology that drive long COVID is relevant to identify therapeutic targets. Current evidence has revealed three main clusters of primary mechanisms [2,9,46,47,48,49,50]: first, virus-related mechanisms that would comprise or damage tissues and organs due to the persistence of the SARS-CoV virus or its components; second, immunoinflammatory mechanisms that imply injuries due to dysregulated immune response, immunopathology, or autoimmunity; and third, endothelial inflammation and immune thrombosis. All these mechanisms may not be mutually exclusive but might coexist and combine their pernicious effects. Some of the current or future mechanism-directed therapies include anti-inflammatories, immunotherapy, immunosuppressants, antivirals (especially in the first 6 months and after reinfection), fecal transplants, vaccines for COVID-19, anticoagulation, or Epstein–Barr vaccine [9].

Because a definitive cure for long COVID does not yet exist, prevention strategies that target modifiable risk factors are needed to curb further increases in long COVID prevalence. Seemingly, public health policymakers would do the right thing considering the prevention of SARS-CoV-2 infections and reinfections as the foundation of the long COVID prevention programs. Some of the factors associated with a higher risk of long COVID or suffering severe symptoms are the pre-existence of medical conditions (such as type 2 diabetes, allergies, history of post-viral fatigue, asthma, chronic lung disease, heart failure, or chronic kidney disease), sex (women), socioeconomic deprivation, multisymptomatic initial acute illness, the severity of initial COVID-19 illness, vaccination status, inability to rest during the initial illness, no antivirals given during initial illness, and recurrent COVID-19 infections [9]. Despite the cumulative knowledge on long COVID, major challenges remain. The proposal for an interdisciplinary research agenda to shed light on long COVID gaps seems reasonable. In addition, the parallels between long COVID and other post-acute infection syndromes suggest that research on long COVID provides an opportunity to understand how infectious agents cause chronic disease [8]. These research efforts on long COVID may also be profitable for optimizing preparedness for future pandemics.

Some potential limitations deserve mention. This bibliometric analysis was based mainly on a single source of data (PubMed). Due to the large biomedical literature coverage of PubMed, it seems reasonable to support that this large database may contain the most COVID-19 scientific publications, including those on long COVID. However, there is no guarantee that this database covers COVID-19 research in its entirety. Other databases such as Scopus or Web of Science could have provided some additional publications. Therefore, critical directions for future research may include elucidating the most proper information sources to track the long COVID research literature, and even exploring the best strategies to combine multiple data sources.

## 5. Conclusions

This bibliometric analysis provides an updated, global, and contextualized perspective of the long COVID literature as of December 2024. The group of long COVID scientific publications represents a relatively low proportion of the total COVID-19 literature, although the magnitude of the cumulative frequency is sufficiently significant to challenge proper information management. This study describes trends and patterns in long COVID publications in the context of COVID-19 research as a whole. Currently, “treatment” and “mechanism” appear to be the most predominant research topics in the long COVID literature. Compared with the rest of the COVID-19 literature, long COVID publications show a distinctive profile, with “case report” and “mechanism” being the most distinguished long COVID research topics. This study also provides a ranking of the leading journals in the long COVID literature production. This study may improve the visibility of long COVID research and contribute to the management of the growing scientific knowledge on long COVID.

## Figures and Tables

**Table 1:** Research topics of long COVID literature compared with other COVID-19 publications, 2020–2024.

	Long COVID Publications	Other COVID Publications	Percentage Point Difference (95% CI)	*p* Value
Total publications, n (%)	8928 (2.3)	380,643 (97.7)		
Research topic ^1^				
Case report, n (%)	3566 (39.4)	79,571 (20.9)	19.0 (18.0 to 20.1)	<0.001
Diagnosis, n (%)	3532 (39.6)	107,969 (28.4)	11.2 (10.2 to 12.2)	<0.001
Forecasting, n (%)	376 (4.2)	27,567 (7.2)	−3.0 (−2.6 to −3.5)	<0.001
Mechanism, n (%)	4279 (47.9)	102,003 (26.8)	21.1 (20.1 to 22.2)	<0.001
Prevention, n (%)	3692 (41.4)	178,048 (46.8)	−5.4 (−4.4 to −6.5)	<0.001
Transmission, n (%)	311 (3.5)	37,659 (9.9)	−6.4 (−6.0 to −6.8)	<0.001
Treatment, n (%)	4515 (50.6)	169,635 (44.6)	6.0 (5.0 to 7.1)	<0.001

^1^ The publications may comprise more than one research topic. Abbreviations: 95% CI: 95% confidence interval.

## Data Availability

Data were derived from the following resources available in the public domain: PubMed database (https://pubmed.ncbi.nlm.nih.gov/). Accessed on 8 January 2025.
